# Climate change’s impact on the nervous system: A review study

**DOI:** 10.34172/hpp.43089

**Published:** 2024-12-30

**Authors:** Mohammad-Reza Sadeghi, Parna Ghannadi, Alireza Lotfi, Hamidreza Ashayeri

**Affiliations:** ^1^Research Center for Pharmaceutical Nanotechnology, Biomedicine Institute, Tabriz University of Medical Sciences, Tabriz, Iran; ^2^Student Research Committee, Tabriz University of Medical Sciences, Tabriz, Iran; ^3^Research Center for Evidence-Based Medicine, Iranian EBM Centre: A JBI Centre of Excellence, Faculty of Medicine, Tabriz University of Medical Sciences, Tabriz, Iran

**Keywords:** Air pollution, Alzheimer disease, Climate change, Dementia, Global warming, Headache, Neurodegenerative diseases, Parkinson disease

## Abstract

**Background:**

Global warming is caused by increased carbon dioxide and other industrial gases, which shift the climate of human habitat and environment, impacting human health globally. In this review, we tried to overview the current knowledge of climate change’s impact on neurological disease.

**Methods:**

A comprehensive search on PubMed, Web of Science (WOS), and Scopus was conducted to find the relevant original studies. Language, sex, age, date, or country of study were not restricted. Included studies report increased Alzheimer’s disease mortality and hospital admission.

**Results:**

This increase was seen from the first day with high temperature to 3-4 days later. Parkinson’s disease (PD) subjects were more vulnerable to high temperatures compared to dementia patients (RR for dementia: 1.29 and for PD: 1.41). Global warming was linked to the increase in the incidence of Tick-borne encephalitis (TBE) (from 0.1% to 5.4%), Japanese encephalitis (OR: 2 when floods occur), and ciguatera fish poisoning (CFP) (RR: 1.62 for each 1 ^◦^C increase per month).

**Conclusion:**

Health-related consequences of climate change are inevitable. The burden of medical problems related to the elderly population (especially the elderly with dementia), infectious diseases, and CFP on the healthcare system will naturally increase. Studying global warming trends could empower us with more precise predictions of the future and better planning to face climate change-related challenges.

## Introduction

 In 1938, G.S. Callendar suggested a link between the increase in carbon dioxide production and the rise in the temperature.^1^ Carbon dioxide and other gases can cause “greenhouse” effects, trapping infrared radiation from the sun and causing a rise in global temperature.^2^ This rise increases the sea level (by melting icebergs) and moisture evaporation (which results in more extreme rainfalls).^3^ The rise in global temperature also causes an increase in the monthly hottest temperature. Alongside global warming, the human population is also growing. Together, these scenarios increase the number of mortalities, morbidities, and health-related outcomes, especially in the elderly age group.^4,5^ Studies show that high temperatures are responsible for 0.46% of disability-adjusted life years,^6^ and over the last ten years, there has been a 600% increase in heat-related cardiovascular disease.^7^ Climate change can increase infectious diseases (due to lower immune response) and non-communicable diseases. The main organs affected by climate change are cardiovascular, Respiratory, and central nervous system (CNS) diseases.^8-11^ Microbes, neurotoxins, air pollutants, and heat stress contribute to the CNS damage.^10,12^ Climate change can affect the pattern of the ecosystem and increase the rates of meningitis and encephalitis. Heat waves are shown to increase the mortality of stroke,^13^ significantly. It has also been proposed that patients taking CNS medications are more likely to suffer from heat waves.^14^ These all indicate a complicated relationship between climate change and CNS-related disease. This review investigates different aspects of brain disease resulting from or affected by climate change.

## Methods

**Figure 1:**
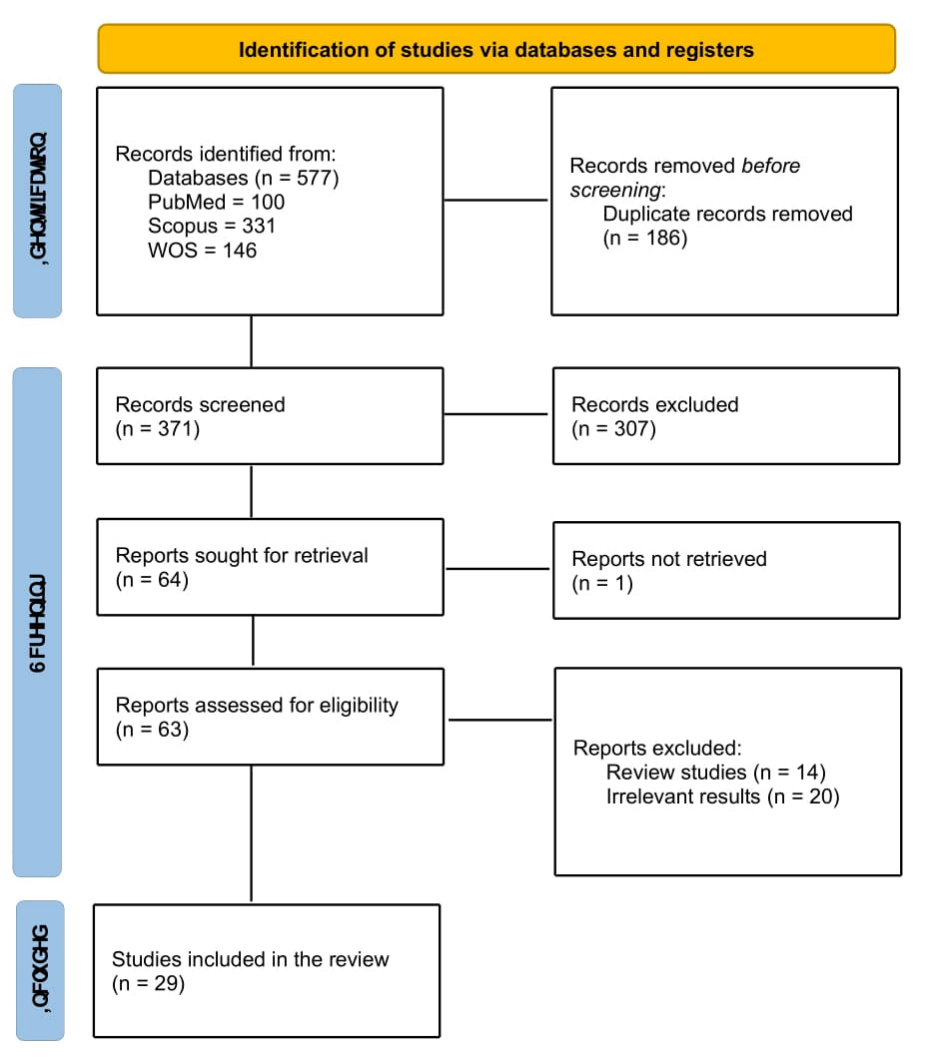
PRISMA flow diagram

 We searched for relevant literature in PubMed, Scopus, and Web of Science (WOS) for the relevant studies. The keywords used were “climate change,” “global warming,” “neurodegenerative disease,” and “nervous system. “The Following search strategy was used in PubMed: ((“neurodegenerative disease”[Title/Abstract]) OR (“nervous system”[Title/Abstract])) AND ((“climate change”[Title/Abstract]) OR (“global warming”[Title/Abstract])). The databases were searched for original studies until the 1^st^ of January 2024. There was no limitation on the Age, sex, language, or country of the study. Original studies investigating the effect of climate change on neurodegenerative disease, CNS-related infections, headaches, and ciguatoxin were included. Excluded criteria were as follows: review studies, in vivo studies, animal studies, book chapters, conference abstracts, and letters. Studies were reviewed by title and abstract, and the eligible ones were included. In the next step, the full text of the studies was reviewed for eligibility, and irrelevant studies were excluded. All the screening steps were done by two authors, and any disagreements and uncertainties were resolved through discussion and the help of a third reviewer. Figure 1 provides the PRISMA flow diagram of our study.

###  Climate change and neurodegenerative disorders

 The hypothalamus is the center of body temperature regulation,^15^ and hypothalamic dysfunction is reported to happen in dementia cases and Parkinson’s disease (PD).^15-17^ The senile community is more likely to develop dementia, making them more vulnerable when facing temperature changes. Senile patients are also at a higher risk of suffering from dehydration, lowering the reserve for body temperature regulation.^18^ Also, drugs taken by them for various diseases (e.g., anticholinergics, diuretics, and dopaminergic drugs) could contribute more to these mechanisms,^19,20^ reducing the elderly population`s compliance against climate change.^21^

 In 2022, Bongioanni et al^22^ gathered the epidemiologic data (prevalence, death, and DALYs) of 3 neurodegenerative diseases (Alzheimer’s disease [AD], PD, and amyotrophic lateral sclerosis) and climate change data from 1990 to 2016. They classify countries based on temperature and rate of temperature increase (warming index) in four regions: high temperature-high warming (HT-HW), high temperature-low warming (HT-LW), low temperature-high warming (LT-HW), and low temperature-low warming (LT-LW). Their analysis shows a higher warming index in countries with lower temperatures. PD was the only neurodegenerative disease affected by the climate change. HT-HW regions increased the PD death rate, and DALLY and a warming index positively correlated with PD prevalence. These results could indicate that neurons behave differently when facing climate change, and their susceptibility to temperature rise differs.

 The temperature range in the Bongioanni et al^22^ study was between 15.9 °C and 29.1 °C in 2016. But does the colder environment always have a protective effect? Another survey by Yin et al^23^ proposes that colder and higher temperatures increase the mortality of neurodegenerative disease, and there is a protective temperature at which the death rate is minimal. Their study had a temperature range of 3.7–24.4 °C and five different climate zones (high alpine zone, temperate continental zone, temperate monsoon zone, tropical monsoon zone, and subtropical monsoon zone). The best protective temperature for dementia was 24-26 °C, and for PD, it was 18.3 °C. They proposed the relative risk (RR) of extremely high temperature (97.5^th^ percentile) mortality in comparison to minimum death temperature for dementia and PD to be 1.29-1.41.^23^ These are in line with previous studies.^22^ PD affects areas in the brain in which neurons are more susceptible to temperature rise, and with the current trend in temperature, the PD burden will likely increase.

 A rise in temperature can increase hospital admissions over some time, starting from the same day and continuing for 3-4 days.^24,25^ From 1998 to 2009, admissions increased at a rate of 4.5% for each 1 °C rise in temperature from 17 °C in the United Kingdom.^25^ These admissions are also predicted to have a 263% increase in 2040 compared to 2009. As the age of the population group increases, the expected numbers also rise. For example, for > 85 years old, 75-84 years old, and 16-74 years old, the increase in 2040 admission was predicted to be 604%, 243%, and 49%.

 Temperature variability also shows an increasing trend because of climate change.^26^ Temperature variability can cause more extreme weather conditions and affect the patients. A cohort study in 2019 by Wei et al^27^ investigates the relationship between dementia-associated hospitalization and seasonal temperature. They report a 12% increase in hospital admissions with every 1.5 °C increase in temperature during summer. They also say temperature variability could increase hospital admissions regardless of season (hazard ratio [HR] of 1.07 for 0.5 °C increase in temperature variability). This study only included patients older than 65, and older individuals are less flexible to temperature variability. Dementia patients are probably at more risk compared to age-matched non-dementia subjects because of higher thermoregulatory disorders incidence in the former group.^17^

 Air pollution, which is a primary culprit for climate change, can directly affect dementia and AD. One retrospective study of over 50-year-old individuals from 1991-2010 revealed a HR of 1.40 for each 10 µg/m^3^ annual increase in N_2_O concentration.^28^ Urban green planning is a strategy to reduce the effects of pollution in urban places and improve health outcomes. A cohort study of 109 688 participants (over 45 years old) was included to evaluate the risk of dementia between locations with different urban green spaces. A lower incidence and better outcome of dementia (HR = 0.86) was reported in locations with > 30% tree canopy.^29^

###  Climate change and migraine

 Migraine is usually a unilateral throbbing headache primarily affecting females and patients in low to mid-income countries.^30^ A case-crossover study from 2000-2007 with 7054 headache patients (2250 migraine and 4803 other types) reveals the odds ratio (OR) of acute headache for a 5 °C increase in temperature to be 1.075.^31^ This study fails to show a lag between temperature rise and headache attacks or a relation between headaches and air pollution. Another study included 102 under 17 years old children accused warm temperatures to trigger migraine attacks in 68.8% of cases.^32^ In 2019, a prospective study of 98 adults was followed for 45 days^33^ and determined that in warm seasons, higher humidity is associated with migraine headaches. These all show a possible increase in migraine attack burden caused by global warming.

###  Climate change and infections

 As the climate changes, the environment of humans and other living creatures alternates. With climate change, more rainfall, floods, increased seawater levels, and less humidity are expected.^3^ These could favor the habitat of vector-borne diseases, as their vectors (e.g., mosquitoes) could grow and reproduce faster and survive longer.^34^

 Temperature variability could affect infectious disease rates as well as neurodegenerative disease. Meningitis is considered a life-threatening inflammation of leptomeninges.^35,36^ Because of the high incidence of meningitis in sub-Saharan Africa, it is called the meningitis belt.^37^ Meningitis tends to cause epidemics in these areas, especially in hot, dry seasons.^38^ Also, wider temperature variability due to a rise in maximum temperature increases the incidence of meningitis with a logarithmic pattern.^39^ Elderly and male populations in Australasia and central sub-Saharan Africa are more susceptible to these changes.^39^ Increasing meningitis surveillance, especially in hot regions affected by global warming, could decrease the disease burden and better control the epidemics.

 Tick-borne encephalitis (TBE) is an important zoonotic viral disease in Asia and Europe,^40^ especially in temperate climate countries.^41^ Tick-bite and dairy consumption are transmission methods of the virus with a summer seasonal pattern for TBE because of the vector’s life cycle.^42,43^ TBE epidemiological pattern (even in a particular country) is affected by geography and climate.^44^ Rising temperatures due to climate change can favor the virus-host environment and increase the incidence of TBE. Most studies on TBE and climate change are in Russian academic literature, focusing on northern territories (especially Arkhangelsk) of Russia.^45-51^ In 2011, Tokarevich et al^50^ hinted at an increase in annual TBE incidence in Arkhangelsk Oblast from 0.1 in 1980-1989 to 5.4 in 2000-2009. Expanding the study period to 42 years, Tokarevich et al^52^ found similar results. They also calculated the normalized difference vegetation index (NDVI) to estimate the environmental changes resulting from climate change and predict the *Ixodid Ricinus*population (a vector for TBE virus).

 Another study by Neh et al^52^ tried to predict the future status of the TBE virus enzootic cycle in 2021-2050 and 2071-2100. They used the TBE monthly average temperature from 1961-1990 with ALADIN-Climate 4.5 and RegCM 3.1 climate models for the task. Their result predicates a 31% (2021-2050) and 50% (2071-2100) rise in the basic reproduction number of TBE virus compared to 1961-1990. Humans are incidental hosts of the TBE virus.^53^ Still, an increase in the enzootic cycle transmission could increase human transmission since the burden of diseases is higher.

 In contrast to previous studies, Palo et al^54^ focus on the relationship between TBE incidence in Sweden and the North Atlantic Oscillation (NAO). They used climate data from 1976 to 2011 but found no relation between TBE cases and summer/winter temperature. Multiple variants (e.g., socioeconomic state or human impacts on the environment) could influence the TBE incidence and affect the results seen in this paper. There is no specific anti-viral treatment for TBE, and with the current direction of TBE incidence, it seems essential to execute vaccination programs in endemic areas.^55^

 Japanese encephalitis (JE) is a mosquito-borne viral disease primarily seen in Asia and the western Pacific.^56^ JE tends to have an outbreak in hot seasons with rainfalls in areas lower than 3000 meters altitude.^57-59^ Climate change can affect JE incidence by different means. A temperature of more than 21-25.2 ^°^C threshold is proposed to increase JE incidence because a larger mosquito population could survive.^60^ Studies investigating the effects of climate change on the JE virus were mainly conducted in China,^60-62^ and India.^63^ A survey by Murty et al^63^ suggests a temperature of 22.8-34.53 ^°^C as the threshold for appropriate mosquito habitat. Humidity and jungle areas are other factors affecting JE epidemiology. JE cases increase when the humidity is more than 65% and NDVI of 150.^61^ Hot weather also increases rainfalls and floods, perfect scenarios for vector-borne disease. In a 5-year study with 370 JE virus-infected individuals, flood significantly increased the odds of JE (OR: 2.00).^62^ Much like TBE, JE could be prevented by vaccination, and routine vaccination of the at-risk population may be needed to avoid disease outbreaks.

###  Climate change and poisoning

 Ciguatera fish poisoning (CFP) is caused by a potent neurotoxin named ciguatoxin from *Gambierdiscus* species.^64^ The toxin accumulates in fish, and humans consuming the contaminated fish get the illness.^65^ Nausea, vomiting, diarrhea, pruritus, paresis, cold allodynia, muscular disorders, paresthesia, and behavioral disorders are the symptoms of consuming ciguatoxin-contaminated seafood.^66-69^ Ciguatoxin binds to membrane voltage-gated sodium channels and causes an increase in neurotransmitter release and cell swelling.^70^ Cook Island,^71^ the French peninsula.^71,72^ the Great Barrier Reef,^73^ the Caribbean Sea, and the Mexican Gulf,^74-76^ were the most common places studied for CFP.

 Studies show that *Gambierdiscus* dinoflagellate survives in a temperature range of 17.5-32.5^◦^C, and a sea surface temperature of ≥ 29 °C is the best temperature for the replication of the organism.^74,77,78^ By analyzing the incidence of CFP in the United States from 2001-2011, Gingold et al^75^ calculate the RR of CFP, calling for a 1 °C rise in sea surface temperature for one month is 1.62. they also showed that even one storm per month increases the CFP calls (RR: 1.11). The authors also predicted a 200%-400% increase in CFP incidence with a 2.5-3.5 °C rise in sea surface temperature. The time lag between sea surface temperature and CPF cases differs from location to location. The reported time lag for Cook Island was 12 months,^71^ and for the French peninsula was suggested to be 13-17 months,^72^ or 32 months.^71^ With the continuous rise in temperature, more heat-resistant species could survive better, and a possibility of change in the dominant species of *Gambierdiscus* microalga is not far.^76^

 Water salinity is another environmental variant that could influence ocean wildlife. The salinity shall increase due to more water vapor,^79^ which can also affect the life habitat of *Gambierdiscus* microalga. By studying the dinoflagellate synthesizing ciguatoxin growth, higher ocean salinity was proposed to increase the number of organisms.^73^ There is no specific antidote or lab test for ciguatoxin, and the diagnosis is mainly based on clinical signs and symptoms.^80^ With the global warming trend, doctors must have a high suspicion of CFP, especially in the summer.

## Discussion

 Air pollution and climate change are shown to affect diseases with different mechanisms. In this review, we showed that dementia and dementia treatment cause thermal dysregulation, putting patients at risk when they face extremely hot or cold weather. Some studies propose a protective temperature for dementia mortality,^22,23^ while others report an increase in dementia-related hospitalization.^25,27^ These findings may seem controversial, but it is also possible that climate change’s relation with dementia-related mortality differs from dementia-related morbidity. It is also noteworthy that the effect of climate change on each neurodegenerative disease is different because the site and neuronal damage in patients differ. PD is reported to be more vulnerable to heat compared to AD. Both diseases are shown to cause hypothalamus dysfunction, but the degree, type, and time of dysfunction occurrence after patients’ primary diagnosis may contribute to the reported difference.

 Global warming will change the habitat of all the creatures on Earth. These changes will benefit some while harming others. As the temperature rises, the environment favors some infectious diseases. TBE and JE incidences will increase because their vectors will have more chances of survival. To counter these phenomena, stricter vaccination programs are needed. There are also new emerging ways to control vector-borne diseases with various tools. Ways to use in this fight are genetic manipulation vectors or the use of Wolbachia parasites to decrease the reproductive output of mosquitos.^81,82^

 CFP is another disease affecting humans, and it will likely benefit from global warming. Included studies show a rise in the growth rate of ciguatoxin synthetizing species as global warming continues its current trend. There was also a lag time between global temperature change and CFP incidence. In the past, CFP has had multiple outbreaks (e.g., six outbreaks in Germany between 2012-2017).^8,3^ This lag time could help us estimate the CFP incidence every year before it happens and be ready for possible outbreaks of CFP.

## Conclusion

 In this study, we tried to overview the possible effects of climate change on neurologic disease. Global warming and climate change will affect many aspects of human neurologic health. The provided data confirms a possibility of a future increase in the healthcare burden of neurologic disease, but the actual intensity of this impact is indefinite. Studying and predicting climate change and finding the relationship between public health and global warming is challenging. Multiple variants (such as race, age, sex, comorbidities, etc.) are involved in human health. There was a vast heterogeneity in methods and results of studies emphasizing the difficulty of researching this subject. A tighter surveillance on climate change and climate-change-related health problems is needed to understand better and predict the future.

## Limitations

 Other neurological conditions can also be affected by global warming, which were not discussed in this review. Increasing temperature can affect neural transmission and affect other neurological diseases, such as multiple sclerosis and myasthenia gravis. This change can cause a worsening in the symptoms and possibly need for an increase in the doses of drugs, which in turn increases the rates of side effects. For future studies, we recommend investigating the effects of temperature rising on the incidence of exacerbations in myasthenia gravis and multiple sclerosis.

## Competing Interests

 None to declare.

## Ethical Approval

 Not applicable.

## References

[1] Callendar GS (1938). The artificial production of carbon dioxide and its influence on temperature. Q J R Meteorol Soc.

[2] Anderson TR, Hawkins E, Jones PD (2016). CO2, the greenhouse effect and global warming: from the pioneering work of Arrhenius and Callendar to today’s Earth System Models. Endeavour.

[3] Zhao W (2020). Extreme weather and climate events in China under changing climate. Natl Sci Rev.

[4] Klein T, Anderegg WR (2021). A vast increase in heat exposure in the 21st century is driven by global warming and urban population growth. Sustain Cities Soc.

[5] Carnes BA, Staats D, Willcox BJ (2014). Impact of climate change on elder health. J Gerontol A Biol Sci Med Sci.

[6] Song J, Pan R, Yi W, Wei Q, Qin W, Song S (2021). Ambient high temperature exposure and global disease burden during 1990-2019: an analysis of the Global Burden of Disease Study 2019. Sci Total Environ.

[7] Al-Kindi S, Motairek I, Khraishah H, Rajagopalan S (2023). Cardiovascular disease burden attributable to non-optimal temperature: analysis of the 1990-2019 global burden of disease. Eur J Prev Cardiol.

[8] Baaghideh M, Mayvaneh F (2017). Climate change and simulation of cardiovascular disease mortality: a case study of Mashhad, Iran. Iran J Public Health.

[9] Joshi M, Goraya H, Joshi A, Bartter T (2020). Climate change and respiratory diseases: a 2020 perspective. Curr Opin Pulm Med.

[10] Ruszkiewicz JA, Tinkov AA, Skalny AV, Siokas V, Dardiotis E, Tsatsakis A (2019). Brain diseases in changing climate. Environ Res.

[11] Van de Vuurst P, Escobar LE (2023). Climate change and infectious disease: a review of evidence and research trends. Infect Dis Poverty.

[12] Ou Y, Wang F, Zhao J, Deng Q (2023). Risk of heatstroke in healthy elderly during heatwaves: a thermoregulatory modeling study. Build Environ.

[13] Zhou L, He C, Kim H, Honda Y, Lee W, Hashizume M (2022). The burden of heat-related stroke mortality under climate change scenarios in 22 East Asian cities. Environ Int.

[14] Layton JB, Li W, Yuan J, Gilman JP, Horton DB, Setoguchi S (2020). Heatwaves, medications, and heat-related hospitalization in older Medicare beneficiaries with chronic conditions. PLoS One.

[15] Sandyk R, Iacono RP, Bamford CR (1987). The hypothalamus in Parkinson disease. Ital J Neurol Sci.

[16] Vercruysse P, Vieau D, Blum D, Petersén Å, Dupuis L (2018). Hypothalamic alterations in neurodegenerative diseases and their relation to abnormal energy metabolism. Front Mol Neurosci.

[17] Diamond PT, Diamond MT (1991). Thermoregulatory behavior in Alzheimer’s disease. J Am Geriatr Soc.

[18] Schols JM, De Groot CP, van der Cammen TJ, Olde Rikkert MG (2009). Preventing and treating dehydration in the elderly during periods of illness and warm weather. J Nutr Health Aging.

[19] Westaway K, Frank O, Husband A, McClure A, Shute R, Edwards S (2015). Medicines can affect thermoregulation and accentuate the risk of dehydration and heat-related illness during hot weather. J Clin Pharm Ther.

[20] Lin MT (1979). Effects of dopaminergic antagonist and agonist on thermoregulation in rabbits. J Physiol.

[21] Grosiak M, Koteja P, Bauchinger U, Sadowska ET (2020). Age-related changes in the thermoregulatory properties in bank voles from a selection experiment. Front Physiol.

[22] Bongioanni P, Del Carratore R, Dolciotti C, Diana A, Buizza R (2022). Effects of global warming on patients with dementia, motor neuron or Parkinson’s diseases: a comparison among cortical and subcortical disorders. Int J Environ Res Public Health.

[23] Yin P, Gao Y, Chen R, Liu W, He C, Hao J (2023). Temperature-related death burden of various neurodegenerative diseases under climate warming: a nationwide modelling study. Nat Commun.

[24] Zhang Y, Ebelt ST, Shi L, Scovronick NC, D’Souza RR, Steenland K (2023). Short-term associations between warm-season ambient temperature and emergency department visits for Alzheimer’s disease and related dementia in five US states. Environ Res.

[25] Gong J, Part C, Hajat S (2022). Current and future burdens of heat-related dementia hospital admissions in England. Environ Int.

[26] Bathiany S, Dakos V, Scheffer M, Lenton TM (2018). Climate models predict increasing temperature variability in poor countries. Sci Adv.

[27] Wei Y, Wang Y, Lin CK, Yin K, Yang J, Shi L (2019). Associations between seasonal temperature and dementia-associated hospitalizations in New England. Environ Int.

[28] Guzmán P, Tarín-Carrasco P, Morales-Suárez-Varela M, Jiménez-Guerrero P (2022). Effects of air pollution on dementia over Europe for present and future climate change scenarios. Environ Res.

[29] Astell-Burt T, Navakatikyan MA, Feng X (2020). Urban green space, tree canopy and 11-year risk of dementia in a cohort of 109,688 Australians. Environ Int.

[30] Li XY, Yang CH, Lv JJ, Liu H, Zhang LY, Yin MY (2023). Global, regional, and national epidemiology of migraine and tension-type headache in youths and young adults aged 15-39 years from 1990 to 2019: findings from the Global Burden of Disease Study 2019. J Headache Pain.

[31] Mukamal KJ, Wellenius GA, Suh HH, Mittleman MA (2009). Weather and air pollution as triggers of severe headaches. Neurology.

[32] Neut D, Fily A, Cuvellier JC, Vallée L (2012). The prevalence of triggers in paediatric migraine: a questionnaire study in 102 children and adolescents. J Headache Pain.

[33] Li W, Bertisch SM, Mostofsky E, Buettner C, Mittleman MA (2019). Weather, ambient air pollution, and risk of migraine headache onset among patients with migraine. Environ Int.

[34] Drakou K, Nikolaou T, Vasquez M, Petric D, Michaelakis A, Kapranas A (2020). The effect of weather variables on mosquito activity: a snapshot of the main point of entry of Cyprus. Int J Environ Res Public Health.

[35] Mace SE (2008). Acute bacterial meningitis. Emerg Med Clin North Am.

[36] Hersi K, Gonzalez FJ, Kondamudi NP. Meningitis. In: StatPearls [Internet]. Treasure Island, FL: StatPearls Publishing; 2024.

[37] Pinilla-Monsalve GD, Llanos-Leyton N, González MC, Manrique-Hernández EF, Rey-Serrano JJ, Quiñones-Bautista JA (2023). Socioepidemiological macro-determinants associated with the cumulative incidence of bacterial meningitis: a focus on the African Meningitis Belt. Front Neurol.

[38] Mazamay S, Broutin H, Bompangue D, Muyembe JJ, Guégan JF (2020). The environmental drivers of bacterial meningitis epidemics in the Democratic Republic of Congo, central Africa. PLoS Negl Trop Dis.

[39] Chen J, Jiao Z, Liang Z, Ma J, Xu M, Biswal S (2023). Association between temperature variability and global meningitis incidence. Environ Int.

[40] Bojkiewicz E, Toczyłowski K, Sulik A (2020). Tick-borne encephalitis - a review of current epidemiology, clinical symptoms, management and prevention. Przegl Epidemiol.

[41] Petri E, Gniel D, Zent O (2010). Tick-borne encephalitis (TBE) trends in epidemiology and current and future management. Travel Med Infect Dis.

[42] Pustijanac E, Buršić M, Talapko J, Škrlec I, Meštrović T, Lišnjić D (2023). Tick-borne encephalitis virus: a comprehensive review of transmission, pathogenesis, epidemiology, clinical manifestations, diagnosis, and prevention. Microorganisms.

[43] Elbaz M, Gadoth A, Shepshelovich D, Shasha D, Rudoler N, Paran Y (2022). Systematic review and meta-analysis of foodborne tick-borne encephalitis, Europe, 1980-2021. Emerg Infect Dis.

[44] Zeman P, Pazdiora P, Benes C (2010). Spatio-temporal variation of tick-borne encephalitis (TBE) incidence in the Czech Republic: is the current explanation of the disease’s rise satisfactory?. Ticks Tick Borne Dis.

[45] Yastrebov V, Rudakov N, Rudakova S (2016). Epidemiology of the transmissible tick-borne infections in Russia. Zdorov’e Naseleniya i Sreda Obitaniya.

[46] Rudakov N, Yastrebov V, Yakimenko V, Rudakova S, Samoilenko I, Poleshchuk E. Epidemiologicheskaya otsenka territorii riska zarazheniya naseleniya prirodno-ochagovymi i zoonoznymi infektsiyami v prigranichnykh regionakh Sibiri [Epidemiological evaluation of contagion risk of the population natural focal and zoonotic infections in the frontier regions of Siberia]. Dal’nevostochnyi Zhurnal Infektsionnoi Patologii. 2015(27):17-9. [Russian].

[47] Kokolova L, Verchovceva L, Gavriljeva L, Kochneva L. Entomological Situation on Blood Parasitic Diseases of Animals in the Yakutia. CABI; 2014.

[48] Sokolova O, Chashchin V, Popova O, Buzinov R, Pasynkova M, Gudkov A (2017). Epidemiological character of tick-borne viral encephalitis extension in the Arkhangelsk region. Ekologiya cheloveka (Hum Ecol).

[49] Burmagina IA, Agafonov VM, Burmagin DV (2014). Characteristics of extreme increase of vector-borne infections in the European North. Kazan Med J.

[50] Tokarevich NK, Tronin AA, Blinova OV, Buzinov RV, Boltenkov VP, Yurasova ED (2011). The impact of climate change on the expansion of Ixodes persulcatus habitat and the incidence of tick-borne encephalitis in the north of European Russia. Glob Health Action.

[51] Tokarevich N, Tronin A, Gnativ B, Revich B, Blinova O, Evengard B (2017). Impact of air temperature variation on the ixodid ticks habitat and tick-borne encephalitis incidence in the Russian Arctic: the case of the Komi Republic. Int J Circumpolar Health.

[52] Nah K, Bede-Fazekas Á, Trájer AJ, Wu J (2020). The potential impact of climate change on the transmission risk of tick-borne encephalitis in Hungary. BMC Infect Dis.

[53] Takahashi Y, Kobayashi S, Nakao R, Kariwa H, Yoshii K (2022). Characterization of tick-borne encephalitis virus isolated from tick infesting dog in central Hokkaido in 2018. Ticks Tick Borne Dis.

[54] Palo RT (2014). Tick-borne encephalitis transmission risk: its dependence on host population dynamics and climate effects. Vector Borne Zoonotic Dis.

[55] Bogovic P, Strle F (2015). Tick-borne encephalitis: a review of epidemiology, clinical characteristics, and management. World J Clin Cases.

[56] Yun SI, Lee YM (2014). Japanese encephalitis: the virus and vaccines. Hum Vaccin Immunother.

[57] Lin CL, Chang HL, Lin CY, Chen KT (2017). Seasonal patterns of Japanese encephalitis and associated meteorological factors in Taiwan. Int J Environ Res Public Health.

[58] Li YX, Li MH, Fu SH, Chen WX, Liu QY, Zhang HL (2011). Japanese encephalitis, Tibet, China. Emerg Infect Dis.

[59] Thakur KK, Pant GR, Wang L, Hill CA, Pogranichniy RM, Manandhar S (2012). Seroprevalence of Japanese encephalitis virus and risk factors associated with seropositivity in pigs in four mountain districts in Nepal. Zoonoses Public Health.

[60] Bi P, Zhang Y, Parton KA (2007). Weather variables and Japanese encephalitis in the metropolitan area of Jinan city, China. J Infect.

[61] Wang L, Hu W, Soares Magalhaes RJ, Bi P, Ding F, Sun H (2014). The role of environmental factors in the spatial distribution of Japanese encephalitis in mainland China. Environ Int.

[62] Zhang F, Liu Z, Zhang C, Jiang B (2016). Short-term effects of floods on Japanese encephalitis in Nanchong, China, 2007-2012: a time-stratified case-crossover study. Sci Total Environ.

[63] Murty US, Rao MS, Arunachalam N (2010). The effects of climatic factors on the distribution and abundance of Japanese encephalitis vectors in Kurnool district of Andhra Pradesh, India. J Vector Borne Dis.

[64] Hoppenrath M, Kretzschmar AL, Kaufmann MJ, Murray SA (2019). Morphological and molecular phylogenetic identification and record verification of Gambierdiscusexcentricus (Dinophyceae) from Madeira Island (NE Atlantic Ocean). Mar Biodivers Rec.

[65] Soliño L, Costa PR (2020). Global impact of ciguatoxins and ciguatera fish poisoning on fish, fisheries and consumers. Environ Res.

[66] Hashmi MA, Sorokin JJ, Levine SM (1989). Ciguatera fish poisoning. N J Med.

[67] Friedman MA, Fernandez M, Backer LC, Dickey RW, Bernstein J, Schrank K (2017). An updated review of ciguatera fish poisoning: clinical, epidemiological, environmental, and public health management. Mar Drugs.

[68] Patel R, Brice NL, Lewis RJ, Dickenson AH (2015). Ionic mechanisms of spinal neuronal cold hypersensitivity in ciguatera. Eur J Neurosci.

[69] Chinain M, Gatti CMI, Ung A, Cruchet P, Revel T, Viallon J (2020). Evidence for the range expansion of ciguatera in French Polynesia: a revisit of the 2009 mass-poisoning outbreak in Rapa Island (Australes Archipelago). Toxins (Basel).

[70] Nicholson GM, Lewis RJ (2006). Ciguatoxins: cyclic polyether modulators of voltage-gated Iion channel function. Mar Drugs.

[71] Zheng L, Gatti CMI, Garrido Gamarro E, Suzuki A, Teah HY (2020). Modeling the time-lag effect of sea surface temperatures on ciguatera poisoning in the South Pacific: implications for surveillance and response. Toxicon.

[72] Chateau-Degat M-L, Chinain M, Cerf N, Gingras S, Hubert B, Dewailly É (2005). Seawater temperature, Gambierdiscus spp. variability and incidence of ciguatera poisoning in French Polynesia. Harmful Algae.

[73] Sparrow L, Momigliano P, Russ GR, Heimann K (2017). Effects of temperature, salinity and composition of the dinoflagellate assemblage on the growth of Gambierdiscuscarpenteri isolated from the Great Barrier Reef. Harmful Algae.

[74] Tester PA, Feldman RL, Nau AW, Kibler SR, Wayne Litaker R (2010). Ciguatera fish poisoning and sea surface temperatures in the Caribbean Sea and the West Indies. Toxicon.

[75] Gingold DB, Strickland MJ, Hess JJ (2014). Ciguatera fish poisoning and climate change: analysis of National Poison Center data in the United States, 2001-2011. Environ Health Perspect.

[76] Kibler SR, Tester PA, Kunkel KE, Moore SK, Litaker RW (2015). Effects of ocean warming on growth and distribution of dinoflagellates associated with ciguatera fish poisoning in the Caribbean. Ecol Modell.

[77] Xu Y, Richlen ML, Liefer JD, Robertson A, Kulis D, Smith TB (2016). Influence of environmental variables on Gambierdiscus spp. (Dinophyceae) growth and distribution. PLoS One.

[78] Llewellyn LE (2010). Revisiting the association between sea surface temperature and the epidemiology of fish poisoning in the South Pacific: reassessing the link between ciguatera and climate change. Toxicon.

[79] Olson S, Jansen MF, Abbot DS, Halevy I, Goldblatt C (2022). The effect of ocean salinity on climate and its implications for Earth’s habitability. Geophys Res Lett.

[80] Friedman MA, Fleming LE, Fernandez M, Bienfang P, Schrank K, Dickey R (2008). Ciguatera fish poisoning: treatment, prevention and management. Mar Drugs.

[81] Wang GH, Gamez S, Raban RR, Marshall JM, Alphey L, Li M (2021). Combating mosquito-borne diseases using genetic control technologies. Nat Commun.

[82] Abraham EG, Cha SJ, Jacobs-Lorena M (2007). Towards the genetic control of insect vectors: an overview. Entomol Res.

[83] Friedemann M (2019). Tropical fish poisonings in Germany 2012-2017 - What is ciguatera?. International Journal of Infectious Diseases.

